# Religion, Spirits, Human Agents and Healing: A Conceptual Understanding from a Sociocultural Study of *Tehuledere* Community, Northeastern Ethiopia

**DOI:** 10.1007/s10943-018-0728-6

**Published:** 2018-11-07

**Authors:** Mesfin Haile Kahissay, Teferi Gedif Fenta, Heather Boon

**Affiliations:** 1 Department of Pharmaceutics and Social Pharmacy, School of Pharmacy, College of Health Sciences, Addis Ababa University, P.O. Box 1176, Addis Ababa, Ethiopia; 2 Leslie Dan Faculty of Pharmacy, University of Toronto, 144 College Street, Toronto, ON M5S 3M2 USA

**Keywords:** Religion, Spirit, Healing, Illnesses, Indigenous

## Abstract

This paper explores the relationship among religion, spirits and healing in the Tehuledere community in the northeastern part of Ethiopia and focuses on how this knowledge can inform primary healthcare reform. The study employed qualitative ethnographic methods. Participatory observation, over a total of 5 months during the span of 1 year, was supplemented by focus group discussions (96 participants in 10 groups) and in-depth interviews (*n* = 20) conducted with key informants. Data were analyzed thematically using narrative strategies. The present study revealed that members of the study community perceive health, illness and healing as being given by God. Many of the Tehuledere people attribute illness to the wrath of supernatural forces. Healing is thought to be mitigated by divine assistance obtained through supplication and rituals and through the healing interventions of nature spirit actors. We found that the health, illnesses and healing were inextricably linked to religious and spiritual beliefs. Our findings suggest that religious and spiritual elements should be considered when drafting and implementing primary healthcare strategies for the study communities and similar environments and populations around the globe.

## Introduction

Although many countries, including Ethiopia, support the principle of a right to health care for all, translating that principle into action is a daunting task for most developing nations. The WHO estimates that more than one-third of the population in developing countries lacks access to biomedical health care (WHO 2011). Focus on biomedical health care as the goal presents a number of challenges for developing nations including: lack of human resources, accessibility, affordability and perceived lack of cultural relevance. This leads to situations where people in developing nations around the globe continue to rely on indigenous healthcare practices along with the biomedical healthcare services (Kassaye et al. 2006).

Jawaid (2015) argue that maintenance and support of indigenous medicine may have a number of advantages. For example, it may be more congruent with the world view of the indigenous people and is likely to be more holistic (i.e., inclusive of prevention and health promotion) than the Western biomedicine which is often focused on treatment of disease, especially in countries with limited healthcare resources. One way to increase the total healthcare resources available within a country is to support the provision of safe and effective indigenous medicine (WHO 2011).

Many scholars have described the important role that religion plays in all cultures, but especially in developing countries. Understanding the perceptions of how to prevent or treat illness would not be complete without exploring the role of religion. Particularly for indigenous communities, religion informs individuals’ understanding of what ill-health is and what health choices are available to them. It provides a broad perspective, beyond the biomedical paradigm, by which to explore a wide range of ways of explaining why one becomes ill, and strategies for re-gaining health (Holt and McClure 2006; Traphagan 2005). Understanding the role of religion and spirituality is the key to providing patient-centered care and promoting culturally appropriate health programs to indigenous communities.

Currently, the government of Ethiopia, through health extension workers, is attempting to provide culturally targeted health messages and interventions that are sensitive to indigenous explanatory models of health, illness and disease. Acknowledging the supernatural and nature spirit agents as well as the social support mechanisms that the indigenous communities associate with healing can help to design community health programs that are more likely to be effective (Healy-Ogden and Austin 2011; Maier and Straub 2011; Malat and van Ryn 2005).

This study focuses on the religious and spiritual contexts of indigenous medicinal practices in *Tehuledere*, a community found in the Amhara regional state of northeastern Ethiopia, and makes recommendations regarding the use of indigenous healing practices as a component of primary health care. By appreciating the “healing” views of the people in these communities, healthcare providers may be able to enhance positive health behaviors.

### The God-Centric Healing Model

The God-centric healing model described by Padela et al.’s exploration of agents and their roles in healing in the Muslim community was a key sensitizing concept that guided the design of this study (Padela et al. 2012). Padela and his colleagues conducted qualitative exploration involving the major ethnic groups within the American Muslim community, a conceptual model emerged of the key agents in healing. The participants related a God-centric narrative wherein God’s will was manifested in the granting of good health or the plight of illness. Moving from illness to health was said to require the individual to seek God’s cure directly through prayer, supplication and recitation of the Qur’an, or indirectly through human agents, and sometimes both. The indirect means of restoring health are found through imams (the individual who is a prayer leader, chief sermon giver and spiritual advisor to the congregation of a mosque), family members, healthcare providers and one’s friends and community. Each agent is viewed as God’s instrument and has various roles within the healing process. Given the importance of religion in the study community, this model provided a starting point to explore the role of God in perceptions of illness, health and healing.

## Methods

The God-centric healing model described by Padela et al.’s exploration of agents and their roles in healing in the Muslim community was a key sensitizing concept that guided the design of this study (Padela et al. 2012; Kleinman 2009 and Helman 2007). The study utilized ethnographic methods (Creswell 2007). Ethnography seeks to understand the social behavior of people living in their natural settings. In this case, a culture-sharing group (*Tehuledere*, the study communities) values, behaviors, beliefs and language were described in relation to religion, spirits, human agents and healing. Given the study’s specific concern with the sociocultural context in which the religious and spiritual meaning of ill-health and healthcare options were constructed, the relevance of the ethnographical method of participant observation was well recognized.

### The Setting

The study was conducted in *Tehuledere Woreda* (district) of South Wollo, northeast of Ethiopia which covers an area of 45,800 hectares with a population of 152,107 (*Tehuledere Woreda* Information Office (TWIO). The capital of the *Woreda*, Haik, is situated 430 km away from Addis Ababa (the capital of Ethiopia). There are a total of 23 kebeles administered by the *Woreda*, of these 19 are rural kebeles, 2 are urban kebeles and 2 are rural town kebeles. Five rural communities were included in the study. The people in this area are members of the Amhara ethnic group and speak Amharic, Ethiopia’s official language. As a predominantly rural *Woreda*, most inhabitants rely on farming. During the study period, the *Woreda* had 2 health centers and 17 health posts. Communicable diseases including malaria, lung infections, diarrheal, intestinal parasites, eye infections, skin disease and rheumatism were the major public health problems in the area (TWIO 2014).

The current religious and spiritual healing beliefs of the community in the study area reflect a mix of pre-Christian Indigenous beliefs, as well as Christian, Muslim and migrant Cushitic Oromo (the largest ethnic group in Ethiopia) influences. The indigenous religion of the Oromo recognized the existence of a Supreme Being and other lesser spirits, namely the “*ayana*” spirits, which were believed to serve as intermediaries between man and the Supreme Being (Hussien 2001). After they migrated from the southern part of Ethiopia, the Oromo people continued to practice their indigenous Cushitic beliefs as well as some pre-Christian animist traditions. Over time, most of these indigenous beliefs were absorbed into Islamic traditions. Our study highlights the importance of understanding the perceptions of spirit/supernatural healing and disease in this community.

### Getting in and Generating Evidence

**Fig. 1:**
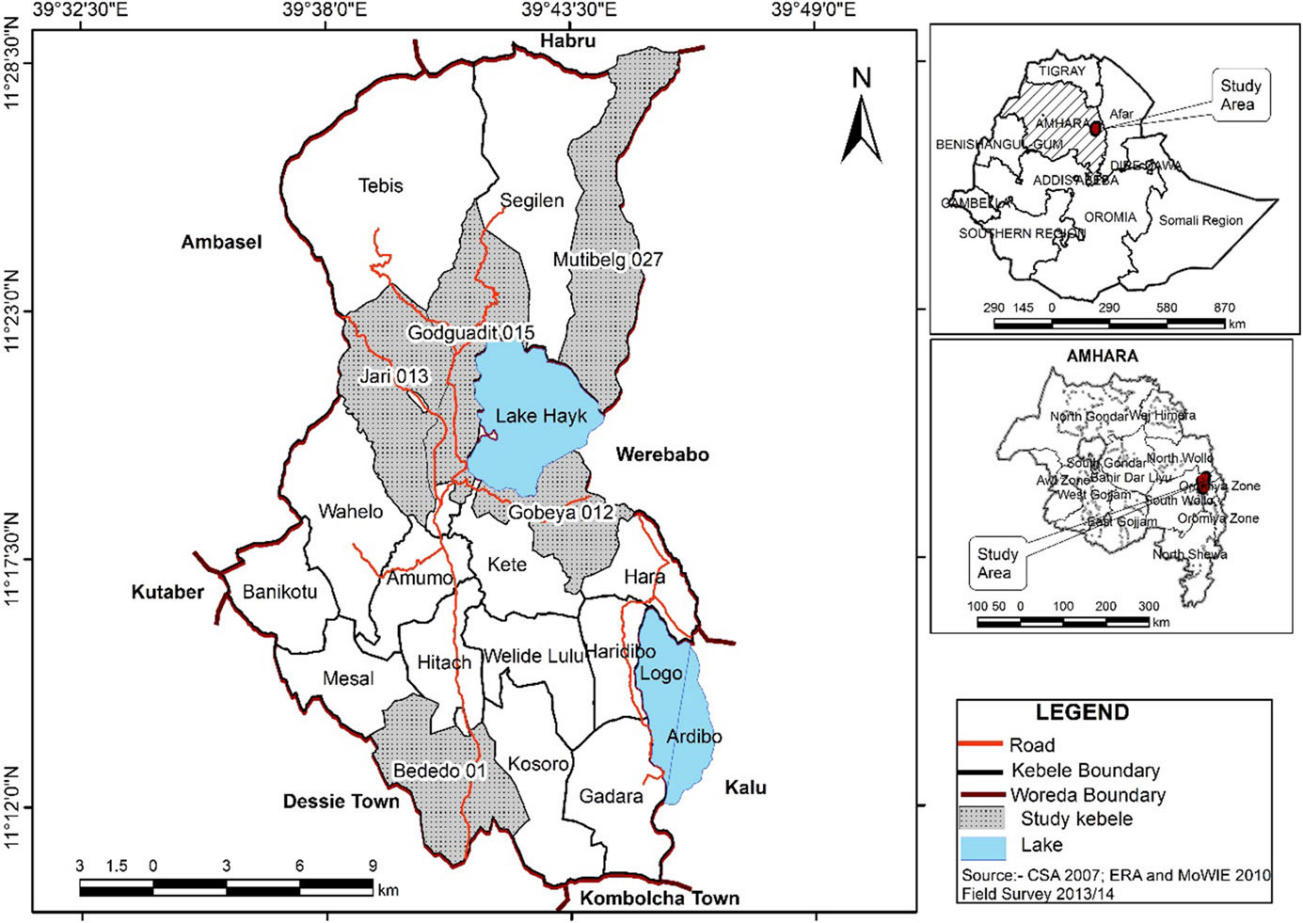
Map of *Tehuledere* Woreda, South Wollo Zone, Amhara Regional State, northeastern Ethiopia, 2014 *Source*: TWIO 2014

Given uniformity of the community in terms of socioeconomic, religion and cultural characteristics, decision was made to choose five *kebeles*, *the smallest local administrative unit*, for the study. The researchers also consulted with the local *Woreda* health extension workers (primary healthcare practitioners) and local elders to select study communities that were accessible and geographically diverse within the five *kebeles*. The five study *kebeles* chosen were: *Gobeya*, *Godguadit*, *Bededo*, *Jari and Muti Belg* (see Fig. 1). Once the five *kebeles* were selected, study participants of focus group discussions and individual interviews were selected in collaboration with health extension workers and local elders. The inclusion criteria for the selection of these participants were: adult of age greater than 30; have lived in the community for 15 or more years; mentally fit, who are verified by the community elders; reported to be knowledgeable about indigenous way of healing.

### Data Collection and Analysis

This ethnographic study was grounded in a participatory observations made by the principal investigator (MHK) functioning as a volunteer heath extension worker in the study area for 5 months between June and November 2013. The investigator was able to observe and participate in local events (for example, ritual ceremonies, merriments, open social events including healing practices) and interact informally with community members. Information acquired from observations and conversations was recorded in field notes, captured in photographs, and when possible voice-recorded for reviewing after leaving the social setting.

In addition, ten focus group discussion sessions (one all-male and one all-female in each *kebele*) were conducted. Study participants were adults over 30 years of age identified by health extension workers in the area to be knowledgeable about local health traditions who were willing to participate in a conversation about their health. Focus groups enabled researchers to conduct group interviews that focused on exploration of the range of opinions and thoughts about religion, spirit and healing. The women’s focus group discussion was meant to allow women to freely and informally discuss their perceptions of religion and healing, without any sociocultural inhibitions (for example, religious prohibitions), which might have inhibited the women from speaking on specific topics if men had been included in the group. The focus groups lasted 1.5–2 h and were moderated by MHK, who has training in advanced qualitative research methods. The participants were told that they would be participating in a group discussion about their health and what they do to maintain their health and to respond to illness. The focus groups included questions about the meaning of illnesses and health, as well as perceptions about their experiences of healing modalities.

Semi-structured key informants’ interviews with 20 interviewees chosen from among the focus group participants were also conducted following the focus groups with a view to obtain a more detailed understanding of the perceptions of ill-health causation, religion and healing. Interviews lasted 1–1.25 h and were conducted in the informants’ private homes or while sitting in public spaces in the villages.

All focus group discussions and interviews were conducted, audio-recorded and transcribed verbatim in Amharic. Early coding, concurrently with data collection, was conducted primarily in Amharic and included multiple readings of the text which were followed by detailed coding and sub-coding schemes around identified issues or themes (Bryman 2012). Two analysts (MHK and another Amharic-speaking team member, TS) immersed themselves in the data by reading and open coding the transcripts independently and developing preliminary codes. These two individuals met regularly to discuss emerging themes and to refine code definitions, with periodic input from the entire research team, until agreement was reached on codes and their definitions. Each transcript was coded line by line, and these codes were organized into higher-order conceptual themes. Sections of original transcripts and key quotes considered to be illustrative of the emerging themes were translated into English to facilitate discussion with the full research team as needed, because one of the research team members, HB, was a non-Amharic speaker. Individual codes and themes were discussed at group meetings until consensus was reached on basic themes and sub-themes across the focus groups and interviews. Finally, the themes were incorporated into a conceptual model of the participants and their beliefs and perceptions in religion, spirits, human agents and healing (Weiss 1994).

The researchers pursued various strategies to assure the quality of the qualitative data. For example, the research findings were shared with research participants and the local research assistants who confirmed the interpretations accurately reflected their perceptions and experiences. The validity of our findings was enhanced by employing different types of triangulation: methodological triangulation (the data collected in the focus groups and the individual interviews were compared and contrasted) and investigator triangulation (multiple members of the research team both in and outside the field participated in data analysis including coding and identification of themes) (Bryman 2012).

All qualitative data from participant observations, in-depth interviews, fieldwork, personal memos and informal conversations were organized using NVivo 10 computer software. Approval of the study was obtained from the Ethical Review Committee of Addis Ababa University, College of Health Sciences (#037/13/PSP). All participants who consented to be included in this study were guaranteed of complete confidentiality, privacy and protection with respect to their character.

### Issues of Reflexivity: MHK Status as an Indigenous Ethnographer

The first author’s (MHK) “native” status offered both opportunities and limitations for the study (Anderson 1992). He approached this ethnographic work as an “Amharic” speaker and tradition bearer, a member of the “Amhara” elite, and also as a senior pharmacy professional. He was able to use existing networks and contacts within the indigenous institutions, including traditional leaders and local health officials, thereby gaining access to a very wide cross section of people. He carefully reflected on how the data collection process influenced his own perceptions, and how other people respond to him. He was also faced with the challenge of being perceived as a powerful individual due to his position as a member of the elite and a senior university lecturer. The use of open-ended questions, as well as informal conversations with informants on topics they themselves raised, was among the ways pursued to mitigate these challenges.

## Findings and Discussion

### Demographic Characteristics of Participants

In total, 96 people participated in focus groups. The number of participants in each focus group ranged from 8 to 12, with a mode of nine. Participants ranged in age from approximately 35–79 years, with a mean of 42 years. Most participants self-identified as Muslims (*n* = 92) and the rest as Ethiopian Orthodox Christians. Most of the participants were married. Just over half (*n* = 53, 53%) reported that they were illiterate, i.e., didn’t read and write Amharic. In total, 20 individuals, 37–75 years old, participated in in-depth interviews (male = 11 and female = 9). They were very similar to the focus group participants in their demographic characteristics. All names attributed to the quotes below are pseudonyms.

### Religion, Nature Spirits, Human Agents and Healing

The importance of religion, reverence of nature spirits and human agents in health and healing were articulated by all study participants. Most ailments were conceived as spirit caused and recommended treatments were likewise enveloped in superstitions, even when they were rooted in biomedicine. We begin by presenting the role that God or Allah is believed to play in health and healing, followed by human agents that respondents believe channel supernatural forces that impact their health and finally the participants’ belief on the biomedicine provided at the local health centers in dealing with some types of illness that are closely linked with spiritual causes.

### The Role of God/Allah

All the participants articulated that their health and well-being are under the direct control of the will of the almighty God/Allah. Many rituals appealed directly to the power of God/Allah and interestingly were celebrated by the entire community regardless of their religious affiliations (Christian or Muslim). The use of amulets, *tsebel*/holy water/and other religious symbols was widespread by participants, and use of symbols commonly had little to do with stated religious affinity and more to do with honoring God/Allah in a general sense.

Almost all of the interviewees made a specific point of thanking God/Allah for their health and well-being throughout the interviews. For example, Fatima, a 56-year-old participant, said:

Many thanks to Allah! I believe in “Allah” and I would pray to Him all day and all night to make our country peaceful and rich, and me and my family healthy … It’s according to Allah, you might be Christian and may not be the same as my beliefs but there is only one Allah, so if He wants you to stay healthy, you stay healthy. So far I have not gone to modern medicine, with the help of Allah I’m healthy, He is my police, my Law, my justice and my court He is everything for me and I can’t say this heals and this doesn’t because everything is in His hands.

This faith was articulated even in situations where it was evident from the physical appearance of the discussants that they were not doing very well. A similar finding was reported in a study of New York City’s Muslim community which highlighted the belief that health was bestowed by God/Allah (Abu-Ras et al. 2008).

People within the study communities widely believe that by observing the laws recommended by God/Allah, every individual could protect himself/herself from possible misfortune. Irreverence to God or Allah is believed across religions and belief systems to cause illnesses, absence of peace and lack of food.

An interesting feature of the study community was that most people celebrated religious ceremonies and followed traditions designed to honor God/Allah that were not based in their stated religions. When the issue of ill-health is raised, there were no religious barriers to seeking healing and prevention from a wide range of religious sources. For example, during our field visit on March, 20, 2014, we observed the “*Tsadiku*”/lit. The saint/festivities. We were invited to one of the villagers’ homes, and the head of the household, 59-year-old Mohammed, explained: “*there are beliefs shared by both Christians and Muslims. For example, ‘tsadiku’ is celebrated by both the Christians and Muslims although this saint is actually a Christian saint. There are people that get cured by the ‘tsadiku’. That is their belief. We don’t work on that day.*”

Many other participants confirmed this widespread example of “borrowing” beliefs, traditions and rituals from a wide range of religions:

We are Muslims, but my husband doesn’t work on St. George’s day (he doesn’t even cut grass for cattle) [Worke, Female, 60)

In our village, there are Muslims who celebrate tsadeku (on March 20^th^) and teklehaimanot (on the 24^th^) every year. We make tela and bukre [local drinks], we bake injera [flat bread] and bread. Thus we have these kinds of traditions that are not just for Christians or Muslims, but for both [Mufti, Male, 65).

Alvarez, the writer in the early sixteenth century, indicated that there was a good relationship between the Christian and Islamic communities in *Wollo*. He was likely referring to the area near present-day *Tehuledere*, in the region previously known as *Ambassel*. His writings describe how those living in the area celebrated all Muslim and Orthodox Christianity religion rituals thought to enhance health, regardless from which religion they belong (Alvarez 1961). This cross-religious relationship appears to have been maintained in the contemporary *Tehuledere* community (Hussien 2001).

During our observation, it was noted that amulets and other symbols (bottle of holy water, religious or prayer books or a cross) from a wide range of religious influences were used for protection from a very diverse range of illnesses. They were believed to ward off illnesses ranging from those supposedly caused by spirits to physical ailments including infections. The belief in the prophylactic and curative power of amulets was very strong. One such example was the cross (traditionally a Christian symbol), which was found widely used within the study population for the healing and prevention of ailments and social health problems, despite the fact that most did not identify as Christians. For instance, we observed Muslim women in *Tehuledere* who adorned themselves with cross-shaped necklaces or tattoos which seem to have no religious connotation to them. Similarly, Assefa (2015) reported that the Cross was generally considered as an amulet and talisman par excellence in Ethiopia.

*Tsebel* or holy water is also found to be one of the most popular healing systems in the study area. Again, although technically based in a Christian belief system, use of holy water was widespread among the entire community.

….. So the holy water heals everyone who uses it. In the eyes of God everyone is equal. Because of our differences in our beliefs, language, and so on, we call ourselves Muslims or Christians. But God did not create us separately. We conspired to bring too many religions. … Because the holy water is given from God, it heals Muslims and the Christians alike. God sees every one with one eye, he does not say black, white, red, bent, straight, tall, short [Priest Admasu, Male, 69].

In our field visit, we observed two, very different, holy water sites: *Bededo* and *St. Stephen*. The *Bededo* holy water healing site is situated at the extreme southwest part of *Haiq* Town. It encompasses two separate springs. The first spring is slightly modified whereby a wall was constructed with two hollow tubes for the holy water to pour down. There is a small wall dividing the space for males and females. Here, the females are more protected by the wall while washing their bodies with the holy water, whereas the males are exposed to the sight of any passerby or observer. People, with different health problems, take off their clothes and sit under the flush of water coming down from the pipes channeling water from the spring.

Ahmed, 59, who brought his sickly wife to *Bededo* holy water site, said that such health problems as itching, swelling of legs and other body parts, headache and *Jinn* attack are resolved through the application of this holy water. Similarly, Kedir, 45-year-old who lives in *Haiq*, said: “Washing with this holy water means securing health.” According to Ahmed, both Muslims and Christians come from the various regions of Ethiopia and use *Bededo* holy water, and they claim that they are cured from their illnesses. As the water hits the holy water users, one can hear a lot of screaming. According to Ahmed, this is an indication of the fact that the evil spirits or any other disease-causing agents are going away, and the individuals are getting relief and refreshment. There is no specific or group to supervise or control the holy water site or to explain the healing procedures. People use the holy water turn-by turn based on mutual understanding and respect for others.

The healing practices of *St. Stephen* were quite different from that of *Bededo.* The holy water is in the compound of St. Stephen Church which is located at the island of Lake Haiq. We observed two categories of clients who needed the holy water healing service at St. Stephen Church: the first category consisted of those people who had minor health problems and who entered the “healing room” by themselves; the second category comprised of those people who had serious or major illnesses and were carried in by relatives and/or friends into the room and then held by force under the flush of the holy water. In both cases, the holy water healer utters words both in Amharic and *Ge*’*ez*^1^ languages, while repeatedly touching the head of the sick with a wooden cross. In the case of the very sick clients, the healer applies both the holy water and the symbolic blow with the holy cross more intensively, and he shouts a lot so that the Satan, *Jinn or Buda* possessing the sick would be gone. The holy water healer asked the disease-causing agent whether it was a Satan, *Jinn*, *or Buda*, and “it replies to his question.” Increasing the intensity, the blow with holy water and the cross, the priest warned the disease-causing agent to dispossess the sick person and disappear. Finally, “the spirit declares that the sick person is free.”

According to one informant, both Christians and Muslims come to the St. Stephen holy water ritual despite the fact that this is technically Christian-based holy water and it is a Christian priest who serves a lead role in the healing practice. Last year, more than 600 patients (Christians and Muslims) were registered in the archive of the church; their religions, places of residence, and their illnesses had been recorded. But according to the deacon, about 10,000 people received the St. Stephen holy water healing services (within a 2-year period, i.e., 2012 and 2013), and they were able to resolve their health problems.

During our field observations, we learnt that health extension workers recognize religious practices such as holy water as helpful in healing Satan, *Jinn or Buda*. In view of such recognition, health extension workers provided religious cleric healers with training on environmental hygiene and medical supplies such as examination gloves. Religious cleric healers on the other hand assist health extension workers to mobilize community members on various community-based health activities including campaigns for vaccinations.

### Human and Nature Spirit Agents: “*Woliy*,” “*Wadaja*” and Healing

In addition to participating in religious rituals from a range of religions, deeply entrenched beliefs in the supernatural world impacted participants’ beliefs about health and illness, regardless of their professed religion. The “evil eye” of the Buda person was a very powerful and widespread example of this. Another example was the *Wadaja*, which is a group ritual ceremony carried out in many houses of Muslim and Christian community members, whereby a person or group with health or any other problems is helped through prayers and songs.

According to many focus groups participants, belief in the *Buda* person, someone who has the power of the ‘evil eye’ and cause illnesses was very common. Buda-caused illnesses were thought to be diagnosed and cured both by indigenous methods often led by *Woliy.*^2^ Christianity and Islam could not disentangle themselves from these highly entrenched ancient beliefs, and thus, rituals spanning both religious traditions, and beyond, have evolved within the study communities to protect against and treat illnesses caused by these forces.

Human agents, such as the *Woliy*, managed some ailments in the study community, perhaps most commonly the *Buda or Ede Tibebat or* Evil eye attack. Regarding Buda management, one male participant stated:

The people that mostly do this are the ones that know ‘kitab’ (Islamic Holy scriptures). They break the egg on the head of patient and read the Asma’aa (Islamic scripture magic). Let alone a child, even an adult with an evil spirit (buda yebelaw) gets cured with this [Ali, male, 62].

Despite the fact that the *Woliy* are not recognized as part of any specific religious establishment in the doctrine, the *Woliy* incorporate reciting Islamic Holy Scriptures in front of people as part of their traditional approach to effect healing.

Many *qolle* or *quteb*, nature spirits,^3^ acknowledged in the study community, were believed to exact tributes in return for physical and emotional security and to deal out punishments for failure to recognize them. Similar to the evil eye, venerating *qolle or quteb*, *wuqabi*, *awlia*, zar and *jinn* spirits were not considered part of either Christian or Islamic religious practices. According to Christian and Muslim teachings, all the supposedly spirit-afflicted illnesses should belong to the family of the Devil. But the Christians and Muslims in the study communities turned to these ancient animistic beliefs without being concerned that I did not appear to fit with their dominant faith.

The idea that the “*Wadaja*” could ward off illnesses and restore health seems to have been the major reason for the persistence and popularity of the ritual among the local population. The *Wadaja* seemed compulsory when a community was affected with communicable diseases (human or animal) and natural calamities such as drought, heavy rain and locust invasion. The ritual was also used for healing purposes when, for example, the family of the sick would summon *Wadaja* experts to conduct a healing session to free the sick from physical or sociopsychological illness and help the patient regain his/her health.

Ahmed, a 55-year-old male participant, had to say:

They [wadajas] say “let’s pray (du’a) with this color of sheep, with this color of goat [to be sacrificed], with this tree, at a particular place”…. if a disease comes, a lot of its bela (bad things) would vanish due to these prayers. God through nature spirit like qolle and qutib would come for us and give us rahemet (economical blessing). Through these prayers [wadaja or dua]) on the said place, with the sacrifices of the said sheep or goat, and the rains come…

In all but one of the focus group, participants noted that the health centers had difficulty assisting with spirit-related ailments like: *qolle or quteb*, *wuqabi*, *mewokel*, *zar*, *buda and jinn*, and that people preferred to visit the cleric healers for this. A participant explained:

If it is jinn /mafatet’, it is believed that the doctor can not cure it thus we take him to the holy water or wadaja will be called. Then the jinn will ‘shout’ and say “I am out” [Kemal, Male, 55]

Another participant added:

There are times when ‘modern’ medicine cannot cure the disease properly. Injections and surgery do not take out the root of the problem. For example, in case a person gets ‘Ebidet’ (madness or Emotional problem) [Kedir, Male, 52]

At times, modern medicine is undermined by the participants’ beliefs. For example, some participants described how they believed some symptoms were wrongly associated with organ failure by the biomedical health workers in the health centers. A male participant said:

What is common in our area is Jinn; but when we go to health centers, they call the condition kidney failure looking at the swell of the victim’s body. We, however, believe that the devil caused the swelling [Kedir, male, 59].

Continuing the description of Jinn attack and its management strategies, Kedir noted,

The major symptom for Jinn attack is swelling. Another thing is that it could lead to a mental illness, making the patient speak out loud. The indigenous treatment is ‘Wadaja’. When you do Wadaja there are things you call out for. The leader of the ritual, usually a sheik, says words from holly Islamic scriptures and calls for the spirit to leave the victim. After that there will be applause …If it is Jinn, the victim sees it in a dream as a human and a monster. At that time, you can see the victim’s body shake. In our culture, there are things you should say in these situations; you say ‘Be’ Muslim’ (In the name of Islam) and sing ‘yaheya keyo’/ritual sing/. Then it will leave the body through the door and scream outside the house. But if it’s not Jinn, the patient won’t be able to see anything so he will be taken to the hospital.

These spiritual beliefs may also contribute to reasons why a patient might avoid using biomedical interventions. For example, some believed that diseases caused by the action of certain spirits like Jinn might be made worse by medication injection. This was because the needle could hurt the disease-causing spirit, causing it to endanger the life of the patient by making him or her unresponsive to other therapeutic or exorcizing procedures. Similar to our findings, Dein et al. (2008) found that the belief in *Jinn* possession was associated with symptoms of mental illness among East London Bangladeshi community particularly when encountering unexplained physical side effects.

The widespread nature of these beliefs and the impact they may have on health-seeking behavior and adherence to biomedical treatments suggests that primary healthcare health service interventions need to incorporate a full understanding of the indigenous beliefs of the community. Biomedical clinicians may need to work closely with religious figures and other indigenous healers, especially when working to support patients with mental illness. Religious clergy may be the first-line emotional healthcare suppliers, especially in areas with active and rich indigenous cures for mental health conditions exist. Rather than ignore or denigrate these traditions, it may be much more effective to identify ways these clergy healers can work with mental health workers to help fulfill the health and spiritual needs of the patient (Osman et al. 2005; Al-Habeeb 2003; Cinnirella and Loewenthal 1999).

Further research should explore the prevalence of mental health issues in individuals who ascribe mental health challenges to suffering by *Jinn*, *qolle or quteb*, *wuqabi*, *mewokel*, *zar* and *buda* specifically among the individuals who look for treatment from faith healers. Creating ways to work synergistically with religious figures and other nature spirit reverence merits further consideration, specifically in connection to distinguishing models of good primary care practice.

### The Emerging Model of Religion, Spirits and Healing

In many ways, the findings from this study are similar to those described in the study that led to the development of Padela et al.’s (2012) God-centric healing model. By and large God/Allah was seen to play a *direct* role in facilitating healing. Most of the discussants in this study engaged in a variety of religious rituals to directly seek healing from God such as begging *Allah* through prayer or *du’a*, and reciting from the Qur’an, celebrating the feast of *Tsadiku*, *using* holy *Kitabs* (magical amulets) and *Tsebel* (holy water). The participants in this study also acknowledged God for his *indirect* healing through the actions of various human agents such as *Woliy* and nature spirits reverence such as *Wodaja* and religious figures. Each agent is viewed as God’s instrument and has various roles within the healing process. In addition, we also found extensive use of indigenous prevention mechanisms for various health-related conditions. What was particularly interesting was how the *Tehuledere* communities had an all-encompassing vision of healing, and often did not distinguish whether a tradition or ritual came from a Christian, Muslim or animistic source. Although the formal religious doctrines attempted to dissuade participants from turning to other forms of spiritual aid, the individuals in this study very commonly participated in rituals from varied sources and did not see any contradiction in doing so.

### Practical Implications

Biomedical service providers in the *Tehuledere* communities will only be effective if they can earn the trust of the community which involves developing strong relationships with both the patients and the traditional healers within the community. Some study participants bemoaned that biomedical providers sometimes did not meet their religious and cultural needs, and voiced worries about their distant interaction style. Such inadequacies in the patient–provider relationship have important ramifications for healthcare utilization and medical services quality. Patient–provider interaction challenges resulting from lack of understanding of patients’ beliefs and values have been found to lead to a poorer quality of care in general including delays in seeking biomedical care, lack of full disclosure of symptoms and concurrent therapies and decreased adherence to treatments (Lee and Lin 2008; Williams and Mohammed 2009). Basic understanding of the culture traditions of the indigenous people, and where possible accommodating and/or integrating care by including indigenous clergy healers in care plans, appears necessary to implementing successful primary care services in the study communities (Chao et al. 2008).

One way to provide a bridge between the religious cleric healers and modern healthcare providers appears to be the health extension workers. Among the roles of health extension workers facilitating integration of the beneficial aspects of religious and spiritual practices and modern healthcare resources is critical for improved health of the local community and optimal use of limited resources in the primary care. It was found that this cooperation of health extension workers, religious cleric healers and modern healthcare practitioners was working well in *Tehuledere* communities.

Our findings suggest that there is a need to improve cultural sensitivity within the biomedical healthcare providers serving the study communities. By understanding the significant actors in health care in these communities for example, God/Allah, *Tsadiqu*, *Tsebel* clergy, *Woliy*, *Qallicha*, we distinguish points of indigenous medicinal services that can be harnessed to improve the well-being of people in the study communities. Given the God-driven perspective of health and healing within this cultural setting, working together with these caregivers can help to spread positive healthcare services messages in ways that are congruent with the religious and spiritual system. Other studies propose that imams and mosques can enhance well-being in a range of different way. For instance, training of imams about tuberculosis brought about mosque sermons prompting expanded location and treatment in Bangladesh (Rifat et al. 2008). In Afghanistan, similar interventions with imams helped to decrease maternal death rates (Mason 2010).

The current Ethiopian indigenous health strategy (the National health strategies with regard to Indigenous Solution reported in March 2015) recognizes the importance of advancing of the useful parts of indigenous medicine including related enquiry and specialized backing. The end goal is to amplify the advantages of indigenous medicine, build new biomedical practices on the foundation of religious and spiritual healing practices. In order for this to be successful, the plans for implementing new essential medicinal services need to be re-evaluated to ensure they fit with the beliefs, needs and interests of the local people (FMOH 2015).

### Study Limitations

The general limitations posed by the ethnography method as a whole also deserve consideration. Perhaps, the most fundamental among these, is the general perceptions about the study itself among the participating communities. Our main collaborators in the study communities were the health extension workers (i.e., government functionaries, by whom we were often accompanied and introduced to various community representatives and members), and this may have impacted local perceptions about the study. Notwithstanding our explanations in regard to the study’s academic nature and specific objectives, some community members could have perceived the study as a government-supported activity. It is not far-fetched, consequently, that the general behavior of informants in both the focus groups and the interviews could be impacted by such perceptions.

The results of this study were based on a sample of participants from *Tehuledere* community. The sample was diverse in terms of age and sex and agroecological condition of the setting. However, the outcomes from this study may not be included all possible opinions and perceptions from the community members.

## Conclusion

The participants in this study clearly articulated that healing, as well as illness and health, is given by God. The healing process takes place by seeking the assistance of the Divine through supplication and scripture-based cures and through nature spirit actors including “*woliy*,” “*wodaja*” *and* “*tsebel*”/holy water. Each nature spirit agent plays several key roles in this process affecting spiritual, psychological and physical health. Exploring ways to incorporate these actors as partners in improving health has the potential to improve study community’s health. Considering their role in the community and relative understanding of the role these agents play, health extension workers were considered to function as bridges between the religious and spiritual healing and modern healthcare system.

Biomedical healthcare practitioners need to be mindful that beliefs about spiritual entities (like *Jinn*) and resorting to supernatural explanations at time of distress are an easily identifiable part of the study communities’ culture. Communication and cooperation between religious leaders and spiritual agents and biomedical service providers should therefore be strengthened for optimal primary healthcare provision.

## Footnotes

1 Ge’ez as a “consecrated” language has also been referred to as the “language of prophecy,” the “tongue of the Angels” and the “first language of Adam and Eve.”

2 Woliy are human agents in Islam that are believed to have the power bestowed upon them by Allah to heal the sickly.

3 Nature spirit, in Tehuledere, is the belief in qolle or quteb, wuqabi, mewokel, zar, buda and jinn spirits that is, venerating certain natural objects as sacred and the abode of spirits. Many natural objects were believed to be capable of mediating between the “supreme deity and the ordinary humans”. The ascription of misfortunes to natural objects pervaded by spirits seems to have been prevalent where by sicknesses, epidemic or contagious diseases and even death were explained in terms of punishment by enraged these spirits.
